# Supplementary figures and images for: Virus transmission via honey bee prey and potential impact on cocoon-building in labyrinth spiders (Agelena labyrinthica)
Source: PLoS One. 2023 Mar 1;18(3):e0282353. doi: 10.1371/journal.pone.0282353 (PMC9977037; doi:10.1371/journal.pone.0282353)

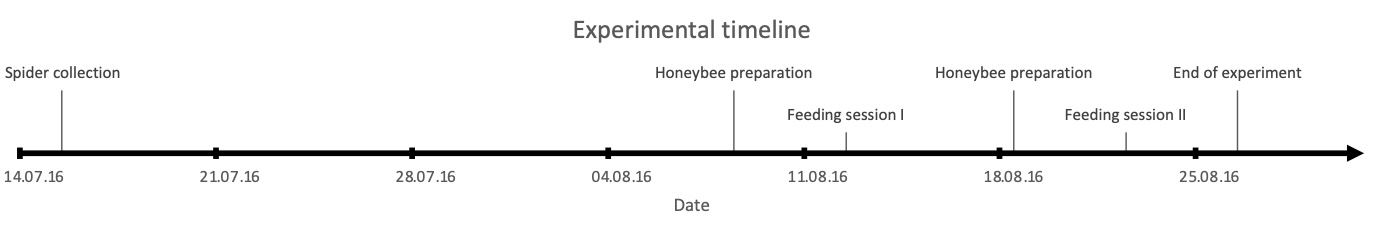

Supplement: S1 Fig — Chronological order of key events starting with the collection of spiders in the field until the experiment got terminated with the freezing of all spider samples at -80°C. (TIF) [file pone.0282353.s001.tif]
